# A Qualitative Study of Pictorial Health Warnings on Malaysian Cigarette Packs: How Do the Adults Understand Them?

**DOI:** 10.3390/healthcare9121669

**Published:** 2021-12-02

**Authors:** Ainun Mardhiah Hamzah, Roslan Saub, Jamaludin Marhazlinda

**Affiliations:** 1 Department of Community Oral Health and Clinical Prevention, Faculty of Dentistry, Universiti Malaya, Kuala Lumpur 50603, Malaysia; dr.ainunmardhiah@gmail.com (A.M.H.); roslans@um.edu.my (R.S.); 2 Oral Health Program, Ministry of Health, Level 5, Block E10, Precinct 1, Putrajaya 62590, Malaysia

**Keywords:** pictorial health warning, cigarette pack, smoking policies, public understanding, adults

## Abstract

The WHO recommended pictorial health warnings (PHWs) on cigarette packs in 2003 to educate and warn the public of smoking effects. Malaysia too has implemented this policy since 2009. This study explored the public’s understanding of the gazetted PHWs depicted on cigarette packs available in Malaysia. A qualitative study using four focus group discussions (FGDs) was conducted among smokers and non-smokers aged 18–40 in Malacca, Peninsular Malaysia. Thematic analyses were performed using the Atlas Ti version 8.0 software. Six themes have emerged reflecting the public’s understanding of the existing PHWs in Malaysia, namely, (i) awareness and exposures, (ii) recall and attention, (iii) perceived goals, (iv) perceived target groups, (v) attitude in understanding, and (vi) knowledge and meaning of PHWs. All participants were aware of the PHWs depicted on legal cigarettes but not seen on most illicit cigarettes. PHWs were perceived to give awareness and warning about the smoking effects targeting smokers and non-smokers. Participants understood the lung and oral health-related images easily than other body parts such as gangrene foot, miscarriages, etc. Besides enforcement on illicit cigarettes without PHWs, policymakers or relevant authorities should emphasize creating relevant and clear pictorial messages in educating the public to avoid confusion affecting the public’s understanding of the PHWs.

## 1. Introduction

Smoking is one of the biggest global public health threats, with 1.1 billion smokers worldwide annually perishing almost seven million smokers and second-hand smokers [1]. Moreover, the entire economic cost of smoking goes beyond the productivity loss due to smoking-attributable diseases/deaths (SADs). The impact also resulted in unimaginable anguish and suffering for the families of those who died due to SADs [2]. Furthermore, SADs, such as other non-communicable diseases (NCDs), have also increased in terms of illness burden and associated health expenditure, both in the Western world and in South East Asia [3,4].

A primary source of concern is that four-fifths of the world’s one billion smokers live in low- and middle-income nations, as is the situation with NCDs [1]. Additionally, a sizable proportion comes from the adult and middle-aged subpopulations (30–69 years old), which is the most productive age and contributes most to their family and country [5]. As a risk factor for six of the top ten leading causes of mortality worldwide in 2015 [1] and four of the top five major causes of death in Malaysia from 2017 to 2018 [6], smoking is a significant cause for concern.

Taking into cognisance of the overall dire situation, the WHO has introduced the MPOWER strategy in 2003, which is a combination of tobacco control policies to reduce the prevalence of active smokers while saving the second-hand (SHS) and third-hand smokers (THS) from the risks and harms of smoking [7,8]. This strategy included a package of six critical and most effective strategies for combating the global tobacco epidemic, specifically, (i) monitoring tobacco use and prevention policies; (ii) protecting people from tobacco smoke; (iii) offering assistance to quit smoking; (iv) warning (educating) the public about the dangers of tobacco; and (v) enforcing bans on tobacco advertising, promotion, and sponsorship plus (vi) raising taxes on tobacco [7]. Whereby, pictorial health warnings (PHWs) on cigarette packs is one of the measures under the ‘W’ (Warn) component of the MPOWER strategy.

PHWs are pictures/pictograms showing the various form of smoking effects, printed in colour, on the front and back panel of a cigarette pack and accompanied with a warning and explanatory text [7]. Studies have shown that compared to text health warnings, PHWs are more memorable, noticeable, can easily attract an individual’s attention and are understood even by the illiterate [9,10,11,12,13,14]. Evidence also shows that combined written and graphic health messages on the packaging of tobacco products are more effective than text-only warnings [15]. Following the ratification with the WHO FCTC in September 2005, Malaysia has implemented PHW simultaneously nationwide, effective from January 2009. As a result, legal provisions related to cigarette sales in Malaysia require the gazetted PHWs to be displayed on individual cigarette packs and cartons. To date, there are 12 gazetted PHW images used in Malaysia that showed neck cancer, lung cancer, miscarriage, stillbirth, premature birth, gangrene, tongue, and mouth cancer [16,17]. Malaysia law requires (i) the presence of any of the gazetted set text and picture (PHWs), (ii) PHWs printed in not less than four colours, (iii) the content of warnings addresses different issues related to tobacco use, (iv) PHW covers 50% of the front and 60% of the back of cigarette packs, (v) PHW printed at the top of the principal display area, (vi) health warning (HW) printed clearly and prominently (clarity), (vii) health warning unobscured (visibility), (viii) health warning text printed in bold, legible font size, style and colour that enhances PHWs visibility, (ix) PHWs in rotation by having multiple messages concurrently or setting a date after which message content changes HW and in a series on an equal number of retail packages, and (x) PHWs appear in principal languages (Bahasa Malaysia and English) [16,17].

Historically, Malaysia started with only six PHWs in 2009 showing the following effects: lung cancer, neck cancer, gangrene, premature birth, miscarriage, and mouth cancer (Figure S1, Supplementary File). The six images were copyright-free, taken from both Thailand and Singapore (three PHWs from each country) as it was perceived to be scarier plus proven to be effective in both countries [18]. The above-mentioned PHWs were chosen after three engagement sessions involving the public and the relevant stakeholders in May 2008 [19]. Moreover, the selection of PHWs to be used in Malaysia and other ASEAN countries was evidence-based. It also underwent a thorough and multilevel selection process before being implemented. Malaysia and other ASEAN countries (e.g., Indonesia, Philippines, Laos PDR, Vietnam) have conducted research in developing the PHWs for their countries. Malaysia, too, has conducted a qualitative study using focus group discussions (FGD) with a large sample size to elicit and gather the public’s consensus (i) of the proposed PHWs, and (ii) a good and effective design [19]. In 2014, another six PHW images consisted of one image of neck cancer, two images of oral cancer, one image of lung cancer, and one each of stillbirth and premature birth was added (Figure S2, Supplementary File). It resulted in 12 gazetted PHW images in Malaysia [16], where six images from 2009 are still continuously being used.

Importantly, understanding a subject helps shape our thoughts and form judgments about it. Studies have shown that higher perceived impacts were observed among those who understood the pictures and messages conveyed by the PHW, and those who possessed adequate health literacy skills [20,21,22]. Hence, these highlights the pivotal role of understanding PHWs towards their perceived effectiveness. Moreover, studies have shown that the understanding of PHWs is contributed and related to individuals: (i) knowledge, experience, and exposure on the smoking effects, (ii) their belief of the impact shown by the PHW, (iii) having adequate health literacy skills, (iv) the way and image plus language used in presenting the PHW, (v) their interpretation of the PHWs, (vi) the roles of mass media, and (vii) the ability to relate any smoking effects anatomically [20,21,22,23,24,25]. The vague understanding of the pictorial contents among most study participants in rural India contributed to the low perceptions of the effectiveness of the PHWs used in the country during the study period [21]. Moreover, in India, earlier in 2011, PHW presented using a symbol or icon was not understood and perceived as least effective by the participants compared to PHWs depicting the X-ray of a diseased lung [22]. In Australia, television advertisements delivering tobacco control messages have contributed to the understanding, acceptance, and impact of PHW, showing smoking effects to significant others [24]. A national study evaluating the effectiveness of PHWs in Australia reported that smokers were perplexed as to how tobacco smoke might affect a bodily component it does not directly contact. As a result, they tended to disregard the health risk by attributing the disease to other plausible causes or co-morbidities such as diabetes [25]. Thus, reflecting the importance of understanding PHWs alongside other tobacco-free initiatives.

As such, this study aimed to explore the public’s understanding of the gazetted PHW’s depicted on cigarette packs available in Malaysia. To our knowledge, no study has assessed the public’s understanding of PHWs in Malaysia after more than a decade of its implementation. The public needs to understand the impact shown by PHWs before an individual can take the necessary actions to quit smoking, ensure they remain a non-smoker or avoid being a victim of secondhand smoke (SHS) or third-hand smoke (THS). Secondhand smoke or passive smoking occurs following involuntary inhalation of ‘the smoke that is exhaled by smokers and that comes from the burning of tobacco products’ [26]. Regardless of the strength of the evidence, SHS is linked to a variety of diseases in children and adults, including sudden infant death syndrome (SIDS), respiratory illnesses, middle ear disease, coronary heart disease, lung cancer, stroke, nasal irritation, odour annoyance, and reproductive effects in women [2]. Third-hand smoke (THS) is defined as the ‘persistent residue of aged cigarette smoke that adheres to dust and surfaces indoors and reemits into the air’ [27]. Both SHS and THS are also public health concerns.

Hence, coupled with the benefits of PHWs being an ideal medium to disseminate tobacco control messages in a multilingual and multicultural society such as Malaysia [21], and the continuous usage of existing PHWs in Malaysia since its implementation, it is critical for this study to be carried out. It is hoped that the study findings could provide an insight into the meaning of PHWs from the public’s perspective and gather the necessary inputs to improve the public’s understanding of the PHW’s to help improve their effectiveness.

## 2. Materials and Methods

### 2.1. Design

This qualitative study using the focus group discussion was conducted in Malacca, Malaysia, between May to August 2019, including recruiting and conducting the FGD sessions. Malacca is a small state (1653 km^2^) located in the southern region of Peninsular Malaysia and populated by approximately one million people, where more than 68% are adults [28]. A qualitative study does not aim to be inferred to the general population but rather to obtain feedback on perceptions, ideas, and contextualize survey findings. Therefore, we opted to use the FGD method to explore the public’s understanding of PHWs [29]. The group interactions dynamic within the FGD method also was more likely to show insight into any issue that might not be present during individual data collection [30]. This state was chosen for this study due to the following three factors:

(i) It was the pioneer state in Malaysia to implement smoke-free laws (SFL) in June 2011 [31], a reflection of its high commitment in the implementation of the anti-tobacco policy;
(ii) although a significant progressive decline in smoking prevalence among adults was seen for Malacca, from 24.2% in 1996 to 16.9% in 2015 [32], ironically, smoking prevalence among youths (adults aged 15–40 years old) in Malacca was high (48.29%) [33];
(iii) Malacca is one of the entry points for cross-border crimes, including illicit cigarettes from the neighbouring country [34], which contributed 52.4% of the cigarette market in Malacca in 2018 [35].

### 2.2. Participants

This study involved adults aged 18–40 years old from all three districts in Malacca. The 40-year-old cut-off point for age was chosen based on the youth age range used in Malaysia at the time of this study [33]. The inclusion criteria were, (i) Malaysian adults aged 18–40 years old residing in Malacca, (ii) who were willing to participate in this study within the study period, (iii) not blind/deaf or mute, (iv) able to understand, speak and write in either *Bahasa Malaysia* or English and, (v) gave their consent. We excluded non-Malaysian citizens. To maintain homogeneity among participants while allowing for divergent viewpoints [36], and given the literature on different findings between smoking status, education level and socioeconomic status [20,25,37], six FGD groups were needed, namely:

- a group of non-smokers,
- a group of professionals:
  - who were non-smokers and had jobs that require advanced education or training, i.e., doctors, dentists, engineers, lawyers, accountants, architects, surveyors, professional technologists and certified technicians, geologists, and town planners.
- four groups of smokers:
  - one group of younger male smokers and ex-smokers (18–25 years old),
  - one group of older male smokers and ex-smokers (26–40 years old),
  - one group of younger female smokers or ex-smokers (18–25 years old),
  - one group of older female smokers or ex-smokers (26–40 years old).

These subgroups were created mainly to obtain a holistic view on the topic from people of different backgrounds through facilitating smooth discussion among people within the groups of homogenous characteristics. Although the ideal number of participants per FGD group is between 5–8 people, due to the sensitivity of the subject and the likelihood that participants may have strong sentiments about it, we limited the group size to five (minimum) or six persons [36]. Thus, the sample size needed was 30 participants. However, considering the possibility of low uptake or no-show of participants; hence the final number of FGD participants after being inflated was 36 people (6 participants per group × 6 FGD groups). For this study, smokers were defined as those who smoked at least once in the past 30 days. We determined the respondent’s smoking status in this study earlier by confirming with them upon their acceptance to participate in this study. Despite the low prevalence of female smokers in Malaysia, we still decided to conduct two FGD groups (similar to the male smoker’s group), considering the possibility of different perspectives among females of different life stages [36] and for more convenient conversation [19], especially concerning PHWs showing pregnancy-related smoking effects. Various attempts and methods were employed to recruit participants for this study. Unfortunately, due to the sudden no-show of male smokers on the day of FGD sessions and no female smokers who were willing and fulfilled the inclusion criteria within the study period, only four FGD sessions were conducted with 24 participants. Therefore, there were no female smokers in our study.

### 2.3. Recruitment

This qualitative study was the second part of three multi-mode studies evaluating the PHWs implementation in Malaysia. Recruitment of participants was carried out through a combination of purposive sampling and multiple techniques, namely, direct approach to the interested party, snowballing, and blasting through social media. Prospective participants were purposely approached in public areas (i.e., bus and taxi stations, shopping malls and tourist sites) and study sites throughout Malacca in the first part of our multi-mode study (Figure S3, Supplementary File 2). The interested person was those encountered during the first part of this study (the PHW compliance study), i.e., the owners, staff, or clienteles in the cigarette point of sales (POS). While collecting data in those POS, we also briefed and invited them to the upcoming FGD. Promotion and invitations to participate in the study were also posted on social media with information regarding its purpose, its procedures; the benefits of participating in the study, and the number to contact if one was interested in participating were made available. The researcher (A.M.H.) approached individuals spotted to be smoking in the public areas and explained to them about this study plus the expected discussion date. The period between the invitations and actual FGD sessions ranged from several days (less than one week) to one month. The contact number of those who are interested to participate was obtained. All prospective participants were briefed about the study, and after obtaining their commitment to participate, the snowballing technique was used. Participants who were directly approached earlier or responded to the social media and committed to attend the FGD were then asked to identify and invite others interested in participating in the study. Lastly, we also disseminated formal invitations to the public through the WhatsApp application via community leaders. The community leaders were the representatives from the Youth Association in this state (*Majlis Gerakan Belia Melaka*)—an umbrella organisation that consists of multiple associations participated by individuals aged 15–40 years old in Malacca.

Due to the low prevalence of female smokers in Malaysia (1.2%) [32], we purposely approached every female smoker encountered in the public, tourism areas, and red-light district in Malacca. The various approaches method was employed to increase the diversity while maintaining homogeneity of study participants. Unfortunately, no female smokers fulfilled the inclusion criteria or were interested in joining the FGD during the study period.

### 2.4. Procedure

This study was approved by the (i) Medical Ethics Committee, Faculty of Dentistry, the University of Malaya [Ref. Number: DF COI903/0003 (P), (ii) the Oral Health Program, Ministry of Health Malaysia [KKM-600-56/7/2 Jld 5 (47)], and (iii) the Medical Research Ethics Committee of the Ministry of Health Malaysia (MREC) [KKM/NIHSEC/P19-1993(5)]. All FGD sessions were held in a hotel meeting room located in Cheng, Malacca, at a meeting point that was accessible (near a highway), comfortable to all participants, and free from any interruptions. Our research team consisted of three members: the researcher (A.M.H.), a note-taker, and a runner (a hired team member who oversaw arrangements of the FGD room, refreshments, preparing the backup equipment and performed other minor tasks). Before each FGD session, patient information sheets (PIS) and written consent forms were distributed and explained to the participants. In addition, demographic information was obtained, followed by a briefing session by the researcher about the objectives and flow of the discussion. Their anonymity and confidentiality were assured, with reporting and future publications according to the pseudonym of their choice.

Participants were divided into their respective groups (as described in Section 2.2) prior to each session. This arrangement ensured a smooth flow of discussion as participants in each group shared the same characteristics. Unfortunately, due to the sudden no-show of several male smokers and no female smokers who fulfilled the study inclusion criteria, only four FGD sessions were conducted. Our FGD participants ranged from four to nine per group, and each session lasted between 60 and 105 min. The FGDs were conducted using the following materials, namely:

- a set of semi-structured guides (Table S1, Supplementary Files),
- a set of 12 gazetted Malaysian PHWs on 12 actual cigarette packs (different brands that were randomly bought at a 24 h convenience store) (Figure S4, Supplementary Files), and
- a digital voice recorder.

Following the ice-breaker session and introductory questions (IQ), the researcher first elicited respondents’ knowledge and understanding of the existing PHWs in Malaysia by asking them the key questions (KQ) without them looking at the actual PHWs. Later, the 12 actual PHWs (Figure S4, Supplementary Files) were shown to the participants to explore their understanding of the PHWs further. Probing questions (PQs) were only used when discussions were stalled. Finally, the discussion was closed with a debriefing session during which key themes were recapitulated and confirmed with participants. Following their debriefing, participants received a monetary honorarium and keepsakes.

### 2.5. Analysis

Audio recordings of the focus group were transcribed immediately after each session. A.M.H. and a professional transcriber (the note-taker) transcribed the audio files verbatim in the discussion language (a combination of Bahasa Malaysia and English). They were then translated to English while ensuring that meaning and content were accurately reflected. The final transcripts were returned to one randomly selected participant from each FGD group for review and confirmation purposes, thus further ensuring the data’s rigour, validity, and trustworthiness [38]. The discussion transcripts were analysed via thematic content analysis [39,40] manually and using the Atlas.Ti Ver 8.0 software by the researcher (A.M.H.). In the first round of manual reading without using the software, the researcher thoroughly and repeatedly read all four transcripts to help familiarise and understand the transcript. The analysis began with an open coding method, in which words, phrases, or ideas uncovered in the transcript were summed up. Disagreements in coding and theme interpretation were resolved via discussion between the researchers. The final coding framework was developed based on the public’s understanding of existing PHWs. Findings were summarised, accompanied by supporting verbatim quotations and tables showing the key points from the FGD.

## 3. Results

The participants’ age ranged from 18–40 years old, with a mean age of 26.9 ± 6.1 years. The majority were Malays (87.5%), educated up to the tertiary level (62.5%), and slightly more than half were male (58.3%). Almost two-thirds (62.5%) were non-smokers and belonged to the low-income group (≤MYR 4000 = USD 970.8). Participants’ professions ranged from homemakers, university students, low manual workers, individuals involved in the business, government servants, and high-skilled professionals (Table 1).

The public’s understanding of the existing PHWs in Malaysia obtained in this study revolved around six themes, namely,

1. Awareness and exposures to PHWs
2. Recall and attention of the PHWs,
3. Perceived goals of PHWs
4. Perceived target groups of PHWs
5. Attitude in understanding the PHWS
6. Understanding, knowledge and perceived meaning of PHWs

### 3.1. Awareness and Exposure to PHWs

Three subthemes have emerged under awareness about PHWs, namely:

(i) General awareness regarding PHW,
(ii) Location of the PHW on the cigarette pack, and
(iii) Presence of PHWs on legal and illicit cigarette packs

All participants were aware of the presence of PHW’s on cigarette packs in Malaysia. A few specifically pointed out its location in the upper part of cigarette packs near the opening. They also correctly mentioned PHWs being present on both the front and back panels of cigarette packs. Interestingly, participants also said PHWs were present on all legal cigarette packs, while most illicit cigarette packs did not have PHWs. Below, in Table 2, are examples of the verbatim quotations (with P denoting the participant).

Two subthemes emerged under exposure to PHWs, which were:

(i) Frequency of exposure
(ii) Sources of exposures

The participant’s exposure to the PHWs varied from frequent, daily to occasional exposures. For example, they reported seeing the PHWs on cigarette packages belonging to themselves, their spouses, fathers, family members, or commonly seeing PHWs when they ‘hang out’ with male friends. Otherwise, PHWs were seen on the cigarette display racks in the following cigarette point of sales: grocery shops, convenience stores, and petrol stations. A few examples of the verbatim quotations are as presented in Table 3 below.

### 3.2. Recall and Attention of PHWs

Regarding gazetted PHWs in Malaysia, our participants recalled PHWs showing cancers (e.g., lungs, neck, and mouth), babies (premature birth and miscarriages), foetus and smoking effects on the neck. Some participants mentioned PHW for liver cancer despite there being no liver cancer image among the 12 gazetted PHWs in Malaysia, while others recalled PHW showing ‘fire in a village’ on illicit cigarette packs. One group did not mention PHWs of the lung effects at all and two groups in this study mentioned and recalled PHW showing the gangrene effect. Only one group quickly mentioned PHWs showing oral cancer, tongue, and lips first instead of other PHWs.

Participants were also able to recall other details about the cigarette packs, i.e., the chemical contents of a cigarette (nicotine, tar, rat poison), the number of cigarettes sticks in an individual pack, the legal age limit for buying cigarettes, and the warning text from the Ministry of Health (MOH) itself. It was also noted that participants from the professional groups (i.e., medicine, engineering, accountancy, and law) recalled more information on the cigarette packs. A few verbatim quotations are as below (Table 4).

### 3.3. Perceived Goals of PHWs

Participants in all four groups repeatedly perceived two points as the goals of PHWs, namely:

(i) to provide awareness to the public, smokers, and non-smokers
(ii) to warn about smoking effects to smokers and future smokers

Impressively, a few participants perceived PHWs implementation as one of the legal, government requirements for licensing and sale purposes. On the other hand, PHWs were also regarded as a medium to:

(i) educate both smokers and non-smokers of the long terms’ risks of smoking
(ii) raise fear among the smokers
(iii) instil awareness to those buying cigarettes, and
(iv) deter one from buying cigarettes.

Table 5 below presents some of the verbatim quotations.

### 3.4. Perceived Target Groups of PHWs

Despite the differences in their smoking status, socioeconomic level, and education level, our participants believed that PHWs targeted both smokers and non-smokers (Table 6). However, for each FGD group, the reasons behind it differed. For example, few groups perceived that PHWs were intended more for smokers as they perceived those smokers would experience the smoking-related health effects first. Contrary to that, some opined that PHWs aimed to provide awareness of smoking effects to smokers and surrounding people, e.g., their spouse and children (second-hand smoking effects).

Besides the two target groups, other participants also mentioned the following target groups, i.e., future smokers, second-hand smokers (SHS), e.g., children of smokers and people who have experienced any of the effects shown by the PHWs. Almost all participants felt that PHWs were not effective at all for smokers. However, most of them perceived PHWs to be more effective towards non-smokers.

### 3.5. Attitude towards Understanding the PHWs

Participants in this study admitted that they had never tried seeking further understanding from any health professional or other sources for any PHW images that were difficult or triggered a question for them. In contrast, some have asked their parents or people around about the images. However, they were either ignored or did not obtain any answers. Among other reasons for not seeking information were that they are not smokers (perceived no reason to bother about it), the PHWs as “unattractive” (not worthy of further inquiries), and they are afraid to ask their parents for fear of being scolded. Otherwise, they acquired the information during health talks delivered by health personnel during their schooling years. Examples of the verbatim quotations are as in Table 7 below.

### 3.6. Understanding, Knowledge and Perceived Meaning of PHWs

Initially, without looking at the actual cigarette packs used in the study, most of our participants claimed to understand all the existing gazette PHWs in Malaysia clearly. However, some of them claimed to understand most PHWs except PHW 4 that shows the gangrenous foot. All participants who did not understand PHW 4 thought that the presented effect was due to complications of diabetes and not from smoking. There was also a perception that PHWs would be difficult to understand for children and those from low socioeconomic and illiterate backgrounds.

Later, upon looking at the 12 gazetted PHWs in Malaysia on actual cigarette packs (Figure S4, Supplementary Files), several distinct subthemes emerged, namely:

(i) PHWs that are easy and difficult to understand
(ii) perceived causative factors for the smoking effects shown by PHWs
(iii) the ability to relate smoking effects anatomically
(iv) variation in perceived meanings of each PHW.

Most participants reported that PHW showing lung cancer (PHW2) was the easiest to understand due to exposure from the education system. Moreover, PHWs showing the smoking effects towards oral health, namely, PHW 3 and 8 (mouth cancer on the lower lips) and PHW 9 (tongue cancer), were easier to understand than the non-oral health-related PHWs (PHW 1,4,5,6,7,10,11,12). They believed that since smoking started from the mouth, the effects could also be starting from there. On the contrary, participants perceived that the PHWs showing cancers (lung-PHW12, neck-PHW7), effects on the vascular system (gangrene foot) and pregnancy-related consequences (miscarriage and premature births) are more difficult to understand due to the inability to relate how smoking could affect the other body parts. Table 8 presents some of the verbatim quotations, including:

In terms of variation in the perceived meaning of PHWs, it was noted that some bizarre comments have emerged, i.e., the perception of PHW showing heart (PHW2), liver cancer and ‘lace’ (PHW 12), besides the perception of ‘operation’ for PHW9. Summary of participants’ perceived meaning and reasons for understanding each PHW is shown in Table 9.

## 4. Discussion

Understanding any health information is vital. It is one of the components in health literacy besides obtaining and utilising the information [41], ultimately leading one to take the necessary health-related actions. Consequently, if the public is unable to understand the health message conveyed by the PHWs, there would be higher chances of them not taking the necessary actions and further lowering opportunities to achieve the intended purposes of PHWs.

Of the limited number of qualitative studies on understanding PHWs, locally and globally, we acknowledged that the number of our participants was relatively low compared to other similar qualitative studies [22,24,25,42]. Of 36 participants required, only 24 participants accepted the FGD invitations. We were unable to recruit any female smokers or ex-smokers who fitted the inclusion criteria. Hence, our first limitation was that only four FGD sessions (involving the non-smokers, professionals, younger male smokers and ex-smokers, and older male smokers) were able to be conducted. The number of participants and sessions should be increased in future studies to achieve data saturation between groups and smokers and non-smokers.

The literature shows a difference in the understanding of pregnancy and baby-related PHWs, in studies that involved female smokers from various age groups and socioeconomic levels [24,25,43]. This may limit our results, particularly opinions of female smokers on the respective PHWs (5,6,11,12). In addition, given our predominantly Malay-Muslim and the lack of non-Malay’s participants in this study, thus some opinions, i.e., attitudes, may be influenced by participants’ sociocultural and religious beliefs. Moreover, recruiting adult participants, especially female smokers, is more challenging, unlike studies involving the population in any organisation or institution (i.e., schoolchildren)—hence lesser chance of obtaining more participants in this study. Nevertheless, this study’s main strength was the first comprehensive study investigating public understanding of existing PHWs after more than a decade of PHWs implementation in Malaysia. Therefore, it is crucial to have this feedback, especially one that involves a range of adult groups. Regardless of the sample number, the given feedback is vital by providing a spectrum of opinions or answers that will be very useful for the policymakers or respective tobacco prevention authorities especially concerning PHWs of cigarette packs.

In this study, all participants were aware of the presence of PHWs on cigarette packs available in the market, similar to findings from an earlier survey of PHWs carried out in Sarawak [44] and a previous cross-sectional study on warning about the harms of tobacco use in 22 countries which includes Malaysia [45]. Indeed, the mention of PHWs presence on all legal cigarettes among our participants indicates compliance of the tobacco industries to the mandatory requirements of PHWs presence on all cigarette packs for consumption in this country.

The most recalled PHWs in this study, those showing cancers and effects on the lung, neck and mouth, premature birth and miscarriages, and smoking effects on the neck, also were akin with the previous studies among adults in Sarawak, India and, among students in Lebanon [21,43,44]. Although this study did not compare findings between subgroups due to the small sample, notable variations in the attention and recall of PHWs were seen. Future studies with a larger sample number could further assess and strengthen these observations. The older participants were noticed paying less attention to the PHWs, and their details were akin to the study [46] among non-smokers, smokers, and patients in London. However, our professional participants noticeably recalled more information and paid more attention to the PHWs, whereas other studies reported their younger participants recalling more [47]. The ability of our professionals to identify more detailed information for the PHWs resonates with other studies where higher awareness of PHWs was associated with higher education levels [47,48].

Regarding attitude in understanding the PHWs, most Malaysian parents, especially the Malays, practice the authoritative parenting style [49] and are not as open-minded as Western parents, especially on sensitive matters such as negative habits (such as smoking), sexual education, etc. Hence, it explained the fear and reluctance among our participants in seeking PHW understanding from their parents. Unfortunately, unlike findings from a study in Europe where a temporary surge of information-seeking over the internet following the introduction of new PHWs [50] was seen, most of our participants reported not attempting to seek further understanding of PHWs, even via the internet. A shocking discovery, considering the increasing pattern in seeking health information via the internet worldwide, including Malaysia [51]. Therefore, future considerations should be given to, (i) inculcate the habit among the public of making efforts to understand any health-related information by using the narrative that understanding health information (whether it is relevant or not relevant to an individual) may save their lives or their loved ones, (ii) encouraging the culture and best practice in seeking health information namely obtaining health information only from reliable sources (e.g., from the relevant subject teachers, health personnel that came to school, the official MoH portal), and (iii) encouraging parents plus caregivers to be attentive to critical questions from their children.

On the knowledge and meaning of PHWs, the most understood PHWs in this study are those related to lung, neck and oral health-related PHWs. Our findings are similar to results in a study for refining the new PHW set in Australia [24]. PHWs showing lung, heart disease, mouth cancer, and throat cancer were among the top ten PHWs with perceived ease of understanding. PHWs displaying smoking effects already known to participants (lung, throat, and mouth) were easily understood and readily accepted, similar to the Australian counterparts in the previously mentioned study [24]. On the other hand, a few of the existing PHWs were not correctly understood despite more than a decade in the Malaysian market. The inability to understand PHWs displaying gangrene, pregnancy-related effects, etc. in this study are examples of the interconnection between a few factors, namely, (i) rationalising the ‘anatomic distance’ of the affected part with where the cigarette was placed during smoking, (ii) their knowledge on smoking effects, and (iii) familiarity with the second-hand smoking (SHS) effects and (iv) perceived belief of the respective smoking effects shown by PHW. Our participants perceived and reiterated their belief that gangrene (PHW 4) is associated with diabetes mellitus (DM) complications. This perception and belief are primarily due to the inability to understand the pathophysiology between smoking and gangrene as shown by the perceived far distance between mouth and foot and familiarity with gangrene as one of the diabetes complications. Correspondingly, the same reasons too were elicited among smokers in Australia who participated in a national study [25].

Furthermore, the poor and misunderstanding observed could also possibly be due to the form and contents of the PHWs. As highlighted by PHWs experts in Australia, higher understanding is achievable if issues regarding the ‘form’ of PHW (wordings, size, graphic elements, placement, and context of PHWs, usage of pictures, and the potential adverse outcome) [23], were addressed. For example, participants in a previous study in India did not understand and perceived PHW using the icon as least effective, instead of other PHWs assessed in the survey (a black and white X-ray of the lungs, etc.) [22]. In contrast, PHWs in Malaysia utilized coloured, real images of smoking effects and followed acceptable photography principles (e.g., contrast, composition, etc.) [52]. However, despite the existing PHWs being fully coloured and printed in the principal languages in Malaysia, some of the PHWs was still not fully understood. These findings emphasized the importance of formulating relatable and clear messages, especially pictorial ones, to avoid confusion among the community. Therefore, besides the opinions and contents from the health experts in the field, it is also crucial to consider the inputs from different target groups at the various stages of developing the contents and messages of PHWs. The respective authorities may want to consider a testing stage too. In this way, the messages of conveyed PHWs have a higher chance of being interpreted clearly and correctly, also relatable to the target groups. Language and choice of wording also matter [23]. It should be easy to understand by the layman, children, and those with low education level and not jargon, i.e., the word gangrene. Hence, adding an explanatory message related to the mechanisms involved for the condition displayed underneath the existing PHWs could achieve a better understanding of it. Nonetheless, the additional explanatory message may consume more cigarette pack coverage than the existing 60% coverage in Malaysia.

In Malaysia, the public was exposed to health-related smoking effects since school age via the national school syllabus, health talks provided by the Ministry of Health (MOH), mass media, and health professionals during their school days. A review supported the roles of the mass media in increasing awareness, knowledge of smoking effects and preventing smoking among young people [53]. Information from the above-mentioned sources may have strengthened our participants’ understanding of smoking effects on the lung, heart, and oral cancer but inadvertently have caused them to believe that gangrene foot was solely due to diabetes. Indeed, a television advertisement prepared by the MOH Malaysia showed gangrene as one of the complications of smoking (Figure S5, Supplementary File). However, the most likely explanation behind this phenomenon was that lack of knowledge of the SHS smoking effect caused the inability to relate and rationalise how smoking caused gangrene, miscarriages, premature babies, stillbirth. Nonetheless, our findings, where participants easily understood and showed salience on the oral health-related PHWs compared to the non-oral health PHWs, were comparable with findings from a current PHWs evaluation study in Australia [24]. Both participants in this study and Australia agreed that the oral-health impacts were due to cigarettes and their smoke touching the tongue and oral cavity.

As for recommendations, future studies should be comprehensive and expanded nationwide similar to the PHW evaluation study in Australia [25]. It is strongly suggested that future research includes participants from all ethnicities in Malaysia for a holistic view of the subject matter. Furthermore, future studies should recruit more smokers, especially female smokers, adolescent smokers, and chronic smokers, from different socioeconomic levels, children, and illiterates. The relevant stakeholders should seriously address issues concerning, (i) the gaps in knowledge of SHS effects, (ii) misperceptions of PHWs showing gangrene and pregnancy-related smoking effects, (iii) issues regarding specific PHWs as highlighted by our participants, (iv) as well as increasing efforts to control illicit cigarette sales. It is vital to raise awareness, correct the misperceptions, and make the public understand the mechanisms behind the identified smoking effects, namely gangrene, pregnancy, stillbirth, and premature birth via suitable mediums, i.e., the education system, the mass and social media. Simultaneously, the respective authorities also may want to replace or improve PHWs that confuse the public. Concerning illicit cigarettes, they often fail to comply with the required health warnings, texts, and labels of a given country [54], and may portray inappropriate messages on the cigarette packs. Therefore, besides causing Malaysia an estimated MYR 5 billion losses from tobacco tax revenue [35] and Malacca being the third-highest state with an increased illegal cigarette trade in Malaysia [55], future monitoring and enforcement activities to control illicit cigarette sales should be reinforced.

Policy-wise, multisectoral collaboration between various stakeholders, i.e., the Ministry of Health, the Ministry of Education, media, respective enforcement agencies and non-government organisations (NGOs) is needed to strengthen future tobacco-free initiatives and control of illicit cigarettes. Public-health wise, both the medical and oral health (OH) counterparts should fortify their efforts in delivering tobacco control messages, specifically to address the misperceptions of smoking effects identified in this study. At the same time, considering the WHO’s policy to integrate brief tobacco interventions into the national oral health (OH) program, specifically the primary OH care [56], we would like to recommend for inclusion of all PHWs used in Malaysia in the antenatal flipcharts, OH talks delivered by the OH personnel, and in the OHP social media contents, at school, chairside in the surgery or during OH promotion activities. Both the medical and oral health sides must work hand in hand to ensure that how those effects occurred is explained to the students, antenatal mothers, and audiences effectively. It should also highlight other smoking effects on oral health, namely periodontal disease, tooth loss and dental caries. Furthermore, policymakers should implement a common risk factor approach for health promotion. Hence, the government could utilise all relevant health and education programmes to educate, raise awareness, and make school-age children, antenatal mothers, and the public understand the whole spectrum of smoking effects.

## 5. Conclusions

This study revealed insights into what and how the public, specifically the adults, understood the existing PHWs in Malaysia. For the adults, understanding the PHWs revolved around awareness and exposures of PHWs, perceived goals and target groups, recall and attention, attitude towards understanding the PHWs, and the understanding, knowledge and meaning of PHWs. For example, the public was aware of the PHWs presence on legal cigarettes in Malaysia, intended to create awareness and warning about the danger of smoking to smokers and non-smokers, while most illicit cigarettes do not have PHWs on them. Besides improving the public’s positive attitude towards understanding the PHWs, the PHWs also need to convey clear and relatable pictorial messages to be well recalled and understood by the public. As such, some images such as lung and oral-health-related images were better understood than images of other body parts; therefore, policymakers or relevant authorities should emphasize creating relevant and clear pictorial messages in educating the public to avoid confusion affecting the public’s understanding of the PHWs. Furthermore, the policy on the rotation of the images also should be further reinforced to avoid desensitization of the same PHWs over a long period. Considering the success of the education system and health talks by the health personnel at school, besides the exposure from mass media in making the public understand some of the PHWs, the same mediums could be officially utilized by the government to improve understanding of the problematic PHWs. It is hoped that in the long run, the combination of these efforts would further strengthen and enhance the effectiveness of PHWs and other tobacco-free initiatives in Malaysia.

## Figures and Tables

**Table 1:** Characteristics of study participants (N = 24).

Socio-Demographic Characteristics	Frequency (n)	Percentage (%)
Age		
18–25 years old	9	37.5
26–40 years old	15	62.5
Ethnicity		
Malay	21	87.5
Chinese	2	8.3
Indian	1	4.2
Gender		
Male	14	58.3
Female	10	41.7
Smoking status		
Smokers	5	20.8
Ex-smokers	4	16.7
Non-smokers	15	62.5
Occupation		
Unemployed	2	8.3
Students	5	20.8
Homemaker	1	4.2
Daily paid worker	1	4.2
Self-employed	2	8.3
Government servant	8	33.4
Private sector worker	5	20.8
Professional status		
Professional	6	25.0
Non-professional	18	75.0
Education level		
Certificate/secondary education	9	37.5
Tertiary education	15	62.5
Household income (MYR) *		
Low income (≤MYR 4000)	14	58.3
Middle income (>MYR 4001–MYR 9619)	10	41.7

MYR = Malaysian Ringgit; * 1 USD = MYR 4.12 (exchange rate at the time of study).

**Table 2:** Examples of verbatim quotations under theme awareness of PHWs.

Subthemes under Awareness	Examples of Verbatim Quotations	Sources
**General awareness**	*“We are aware of its (PHWs) presence.”*	(All participants, P1–P24, Non-smokers, ex-smokers, smokers)
**Location of the PHW**	*“Yes, I have seen them (the health warning pictures). It’s located at of cigarette pack-near the opening.”* *“It (PHWs) was displayed at the front and back of the cigarette box.”*	(P3, Female, Non-smoker) (P16, Male, Ex-smoker)
**Presence of PHW on legal and illicit** **cigarette packs**	*“Nowadays, all legal cigarettes have PHWs printed on them”* *“Some illicit cigarettes do have them (PHWs), but the majority (of illicit cigarette) does not contain any PHW”*	(P21, Male, Smoker) (P24, Male, Smoker)

**Table 3:** Examples of verbatim quotations under theme exposure to PHWs.

Subthemes under Exposure	Examples of Verbatim Quotations	Sources
**Frequency of exposure**	“*I definitely see it every time I open the cigarette pack.”* *“I always see it (PHW). In fact, I just saw it before I came here. I saw on my husband’s cigarette pack that was on the table at home.*	(P21, Male, Smoker) (P6, Female, Non-smoker)
**Sources of Exposure**	“I’ve seen it (PHW) before, on the cigarette packs displayed in the 7E***** (a type of nationwide franchised convenience store or cigarette point of sales) or being held by male friends when we hanged out.” *“It is the same for me. I only saw it (PHWs) on the cigarette packs. The thing is, I used to see it more often when my husband is a smoker. I rarely see it now because my husband quit smoking years ago “*	(P12, Male, Non-smoker) (P14, Female, Non-smoker)

**Table 4:** Examples of verbatim quotations under theme recall and attention of PHWs.

Subthemes under Recall and Attention	Examples of Verbatim Quotations	Sources
**PHW images**	*“There’s a lot of pictures …Foetus, lung, mouth cancer, liver cancer and a damaged lung.”* *“On the pack, I’ve seen picture of the foot.”* *“There was image of the tongue and mouth”*	(P15, Male, Non-smoker) (P19, Male, Ex-smoker) (P23 & P24, Male, Smoker) (P17, Male, Ex-smoker)
**Unique images on illicit cigarette packs**	*“I remembered one picture (PHW) from a neighbouring country. It stated that smoking could set fire to the whole village.”*	(P11, Female, Non-smoker)
**Other details on cigarette packs**	*“About the warning that I’ve noticed was stating that it was from the Ministry of Health.”* *“There’s the warning word, the danger of smoking, tar and constituents in a cigarette, e.g., the rat poison etc.”* *“I remembered that there was something about the contents in gram (quantity), the number of cigarette sticks.”* *“There was the warning word, something on 18 years and above the legal age of purchasing cigarette), something about being fined and other sorts of warning.”*	(P7, Female, Non-smoker) (P8, Male, Non-smoker) (P24, Male, Smoker) (P17, Male, Ex-smoker) (P13, Female, Non-smoker)

**Table 5:** Examples of verbatim quotations under theme perceived goals of PHWs.

Subthemes under Perceived Goals of PHWs	Examples of Verbatim Quotations	Sources
**To give awareness**	*“For me, the pictures are two-pronged approach (awareness and educating). It is informing the danger of smoking to the existing smokers and what is expected to the future smokers.”*	(P8, Male, Non-smoker)
**To warn about smoking effects**	*“Giving warning and knowledge of smoking effects on smokers. For some smokers, perhaps they just followed their (smoking) friends without knowing the effects. But, with the picture, maybe they would know and quit smoking.”* *“It’s like a warning of future health effects to the smokers.”*	(P15, Male, Non-smoker) (P10, Male, Non-smoker)
**Other goals of PHWs**	*“PHWs were displayed because it is one of the conditions imposed by the government to the licensed cigarette, to display the warning* *pictures.”* *“The purposes are to give warning, to scare people and motivate smoking cessation.”* *“To make people realise, feel sorrow, or repent to those who want to buy the cigarettes. So, when we looked at cancer pictures on the box, it would deter us from buying it.”*	(P20, Male, Smoker) (P22, Male, Smoker) (P19, Male, Ex-smokers)

**Table 6:** Examples of verbatim quotations under the theme perceived target groups of PHWs.

Subthemes under Perceived Target Groups of PHW	Examples of Verbatim Quotations	Sources
**Smokers, Non-smokers and Public**	*“It was targeted to all…to smokers and the public.”* *“To me, those images were intended to scare the smokers, but it’s not scaring them at all. Instead, it managed to scare us, the non-smokers...ha-ha”* *“I think PHW is targeting more on smokers. Because they would be the first one affected.”* *“Children too benefited from the PHW. They would say that their (smoking) parent’s lung would be like the picture. Just like what my nephew said to my brother, and indeed later my brother quit smoking.”*	(P9, Male, Non-smoker) (P21, Male, Smoker) (P17, Male, Ex-smoker) (P11, Female, Non-smoker)
**Future smokers and Second-hand smokers**	*“It (PHWs) is targeted to smokers and to anyone who is planning to smoke in future.”* *“To give awareness to smokers, and for non-smokers, to show the possible smoking effects, e.g., to those around them, their wife and child.”*	(P8, Male, Non-smoker) (P2, Female, Non-smoker)

**Table 7:** Examples of verbatim quotations under the theme attitude towards understanding of the PHWs.

Subthemes under Attitude in Understanding PHWs	Examples of Verbatim Quotations	Sources
**No effort/No attempt to understand at all**	*“We made no effort to understand it.”* *“I did not attempt to understand it because it was unattractive.”*	(P21, P22, P23, P24, Male, Smokers) (P13, Female, Non-smoker)
**Attempted to seek understanding from parents**	*“My father is a smoker...I’ve tried asking him once about the relationship between the images shown on the cigarette box with smoking. But it seemed like he didn’t know anything about it and didn’t bother to explain it to me.”* *“I’m afraid that if we asked an explanation about it from our parents, we would be scolded.”*	(P3, Female, Non-smoker) (P17, Male, Ex-smoker)
**Indirect attempt to seek understanding**	*“I have never asked an expert to understand the PHWs. But I have attended health talks at school before. Those talks informed us about the danger and effects of smoking, including effects on premature babies,* etc. *So that’s how I understand all this.”*	(P2, Female, Non-smoker)

**Table 8:** Examples of verbatim quotations under the theme understanding, knowledge and meaning of PHWs.

Subthemes under Understanding, Knowledge and Meaning of PHWs	Examples of Verbatim Quotations	Sources
**Discussion without looking at the actual cigarette packs**		
**General**	*“I understood the meaning of PHW.”* *“I understand the other images, but I didn’t understand the foot image.”* *“It depends on the level of education. From my observation, those who didn’t go to school might not understand the pictures.”* *“This one look like diabetes (PHW 4).”*	(P24, Male, Smoker) (P16, P17, P18, P19, P20, Male, Smokers and Ex-smokers) (P12, Male, Non-smoker) (P22, Male, Smoker)
**Discussion after looking at the actual cigarette packs**		
**PHWs that are easy and difficult to understand**	*“The lung pictures (easiest to be understood) because we have already been informed (taught) at school about it.”* *“I could understand PHWs related to the mouth (PHW 3,8,9). Smoking started from the mouth. Hence, the effects could first be seen there.”* *“These pictures (PHW 4,5,6,7,10,11-gangrene, neck cancer, pregnancy, stillbirth, premature birth) are difficult to understand.”* *“Couldn’t understand this too (PHW 5), if we didn’t look at it properly, one might think that the baby is still in the womb.”*	(P17, Male, Ex-smoker) (P8, Male, Non-smoker) (P1, Non-smoker) (P15, Male, Non-smoker)
**Perceived** **causative** **factors for the smoking effects shown by PHWs**	*“For PHW 11 (premature birth), maybe the mother herself is having problems that caused this effect.”* *“This (PHWs of cancer), it could be due to dietary factor.”* *“Number 4, people would think caused by diabetes.”*	(P14, Female, Non-smoker) (P24, Male, Smoker) (P15, Male, Non-smoker)
**Ability to relate smoking with smoking effects anatomically**	*“I didn’t understand, we smoked a cigarette in the mouth region. Why does it affect the foot?* *“The one I couldn’t understand is the foot. How does smoking affect the foot?”* *“I can still understand number 12 because the ciga-smoke that we inhaled would go to the lung*.	(P5, Female, Non-smoker) (P12, Male, Ex-smoker) (P3, Female, Non-smoker)
**Variations in perceived meanings of PHWs.**	*“I don’t even know that this (PHW 12) is a lung. I thought it was a blackened tongue or else.”* *I guess number 9 is showing an operation (not a smoking effect).* *“The lung shown at the front and at the back is the picture of the liver cancer (PHW 12).”*	(P4, Female, Non-smoker) (P1, Female, Non-smoker) (P15, Male, Non-smoker)

**Table 9:** Summary of participants’ perceived meaning and understanding of each PHW.

PHW	Actual Meaning	Perceived Meaning of Each PHW
1	Health effects: larynx *	As a direct effect of smoking Neck cancer Effect on the trachea
2	Health effects: lung *	As a direct effect of smoking Lung cancer Damaged, blackened lung Heart problem
3	Health effects mouth disease/oral cancer *	Smoking effect on lips Mouth cancer
4	Health effects: vascular system/gangrene *	Complication/effects of diabetes Not related/not caused by smoking
5	Pregnancy *	Second-hand smoking effects (SHS) Complications of maternal problems or other factors, e.g., diet, medications Intended only for female (smokers and non-smokers)
	
6	Neck cancer **	Neck cancer Thyroid disease
7	Mouth cancer **	The first site that would be affected by smoking Lip cancer Some could not identify the location (which body part)
8	Tongue cancer **	Operation Routine dental check-up
9	Stillbirth **	Second-hand smoking effects (SHS) Complications of maternal problems or other factors e.g., diet, medications Intended only for female (smokers and non-smokers) Abandoned babies
10	Premature birth **	
11	Lung cancer **	Resembles a lace (the black striaes on the lung images) Liver Blackened lung Unclear image, difficult to understand

Note: Please Refer to Figure S4, Supplementary Files for the number and PHW images. * According to the WHO FCTC Database, ** According to the text printed under the PHWs.

## Data Availability

The data presented in this study in this study are only available on request basis from the corresponding author. It was not publicly available due to privacy and ethical issue.

## Supplementary Materials

The following are available online at https://www.mdpi.com/article/10.3390/healthcare9121669/s1, Figure S1: The first PHW images used in Malaysia starting from 2009 title, Figure S2: The additional PHW images used in Malaysia starting in 2014, Figure S3: Distribution of study areas covered in the Phase 1 of this study where participants also were directly approached for FGD, Figure S4: Actual cigarette packs showing 12 gazetted PHWs in Malaysia used during the FGD sessions, Figure S5: Evidence of previous television advertisement prepared by the Ministry of Health Malaysia showing gangrene as one of the smoking consequences, Table S1: Script guides for the focus group discussions (FGD).
