# Supplementary material for: A Qualitative Study of Pictorial Health Warnings on Malaysian Cigarette Packs: How Do the Adults Understand Them?
Source: Healthcare (Basel). 2021 Dec 2;9(12):1669. doi: 10.3390/healthcare9121669 (PMC8700954; doi:10.3390/healthcare9121669)
Supplement: Supplementary file 1 [file healthcare-09-01669-s001.zip › healthcare-1468563-supplementary.pdf]

Supplementary Files

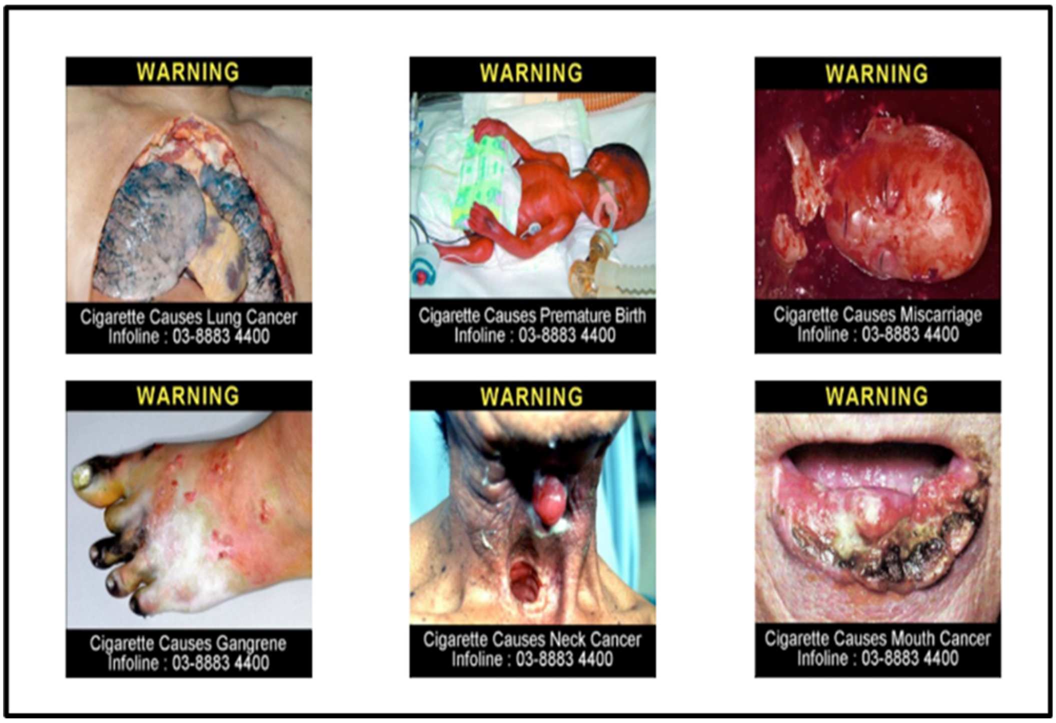

Figure S1: The first PHW images used in Malaysia starting from 2009

Source : *HYPERLINK "https://tobaccolabels.seatca.org/malaysia-graphic-health-warnings/" https://tobaccolabels.seatca.org/malaysia-graphic-health-warnings/*

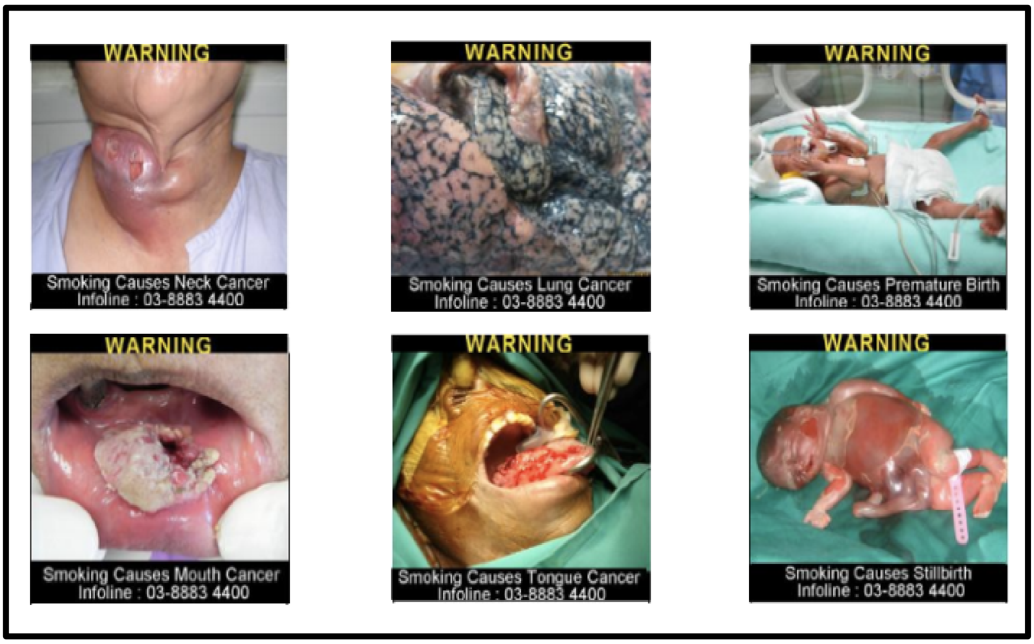

Figure S2: The additional PHW images used in Malaysia starting in 2014

Source : *HYPERLINK "https://tobaccolabels.seatca.org/malaysia-graphic-health-warnings/" https://tobaccolabels.seatca.org/malaysia-graphic-health-warnings/*

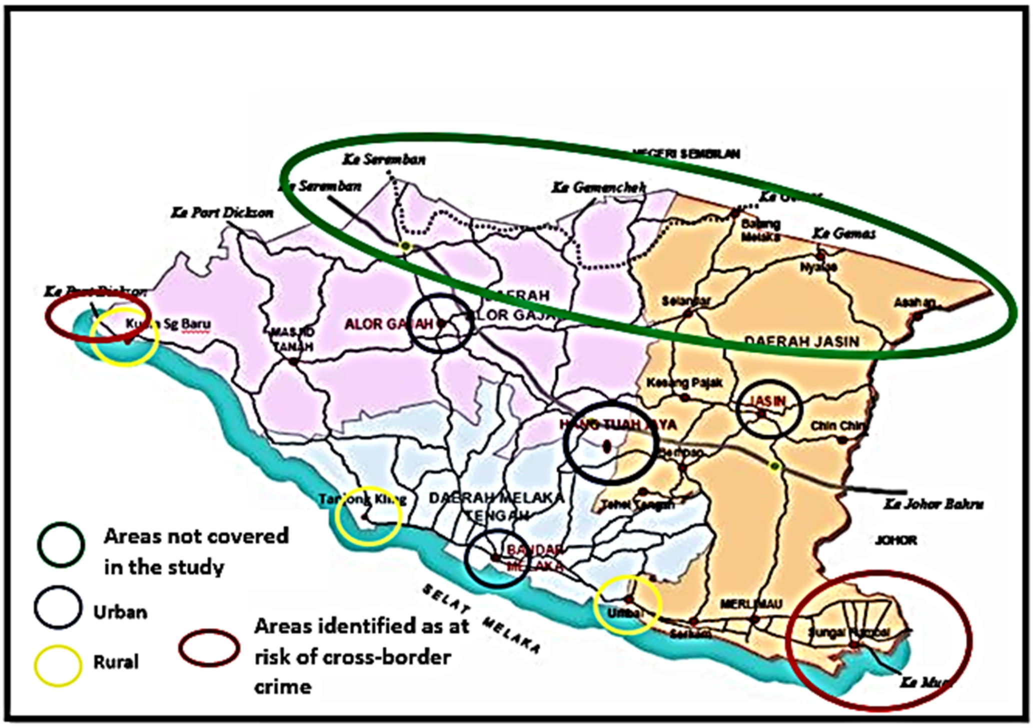

Figure S3: Distribution of study areas covered in the Phase 1 of this study where participants also were directly approached for FGD

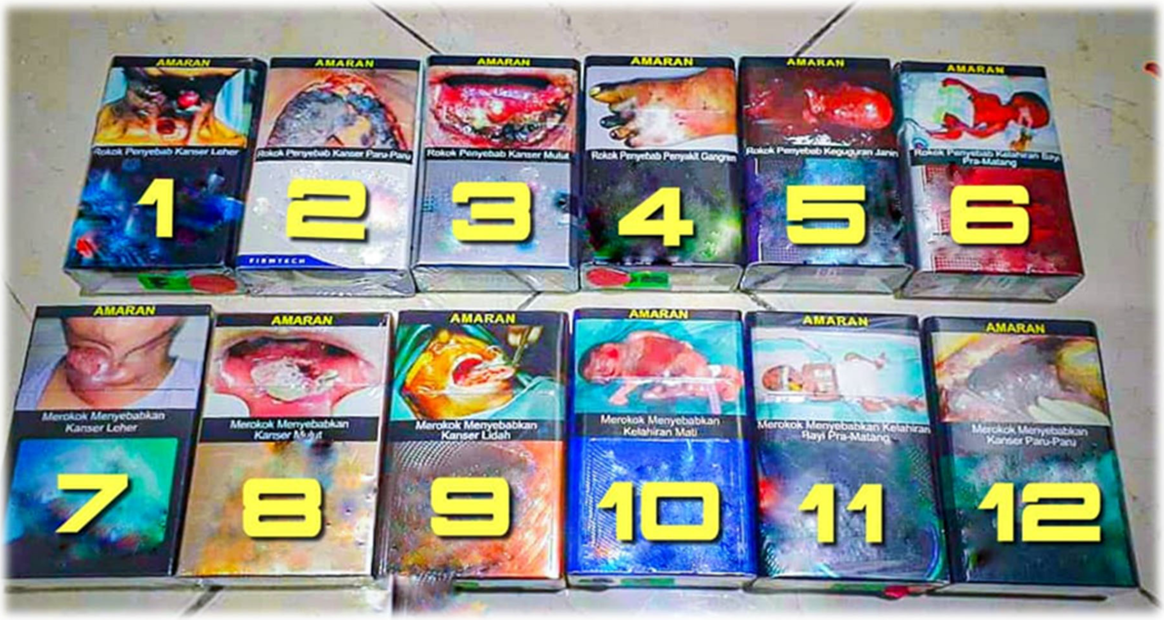

Figure S4: Actual cigarette packs showing 12 gazetted PHWs in Malaysia used during the FGD sessions

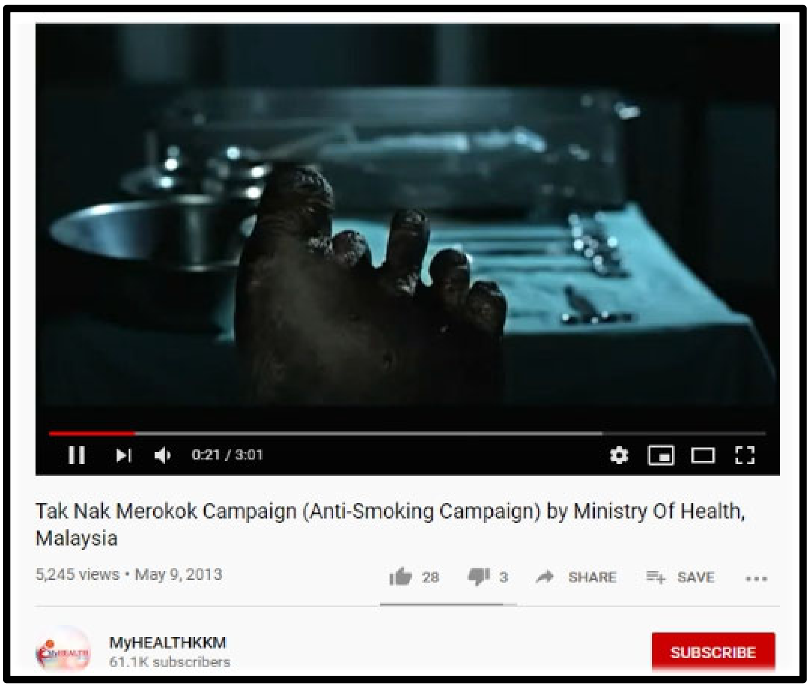

Figure S5: Evidence of previous television advertisement prepared by the Ministry of Health Malaysia showing gangrene as one of the smoking consequences

Source: Tak Nak Merokok Campaign (Anti-Smoking Campaign) by Ministry Of Health, Malaysia, Accessed from https://www.youtube.com/watch?v=4O2xw_kpPJU

Table S1: Script guides for the focus group discussions (FGD)

**Questions***		**Rationale/comments**
**KQ/ IQ**	What do you know about health warning pictures on the cigarette boxes/ packs in Malaysia?	• This key question (KQ) was posed to allow for further introductions to the participants and to gradually direct the discussion toward the study topic.<br>• It was also asked without participants looking at the actual PHWs on cigarette packs.
**PQ.**	Have you ever seen a health warning picture on a cigarette box before?	The probing questions (PQ.) were only used when discussions are stalled
**PQ.**	At which part/s of cigarette box did you see the health warning picture?	
**PQ.**	How often do you see this picture?	
**PQ.**	When was the last time you saw this picture?	
**PQ.**	Have all the cigarette boxes that you've ever seen before contain health warning pictures?	
**PQ.**	Do you remember what information is there in the pictures? Why do you say that?	

Questions*		Rationale/comments
KQ.	In your opinion, what is the purpose of the health warning pictures?	
**PQ.**<br>**PQ.**	Do you know the purpose of the pictures shown on the cigarette box? Why do you think so?<br>To whom was the message intended? Why do you say that?	The probing questions (PQ.) were only used when discussions are stalled
KQ.	Can you share what you understand about the health warning pictures on the cigarette box?	• This key and the accompanying probing questions were asked without showing the set of 12 gazetted Malaysian PHWs on 12 actual cigarette packs
**PQ.**<br>**PQ.**<br>**PQ.**	Based on your experience of seeing the health warning pictures on the cigarette boxes and from your understanding, what are the pictures trying to convey to us? Why?<br>Are there any educational or warning elements? Why do you think so?<br>Which picture is easier to understand and difficult to understand? Why?	The probing questions (PQ.) were only used when discussions are stalled
**KQ.**	From your understanding, what do the health warning pictures in front of you try to convey?	A set of 12 gazetted, PHW images for Malaysia's cigarette packs were displayed to the participants as reference points for the probing questions.
**PQ.**<br>**PQ.**<br>**PQ.**	Can you share the meaning of these pictures according to your understandings? Why do you say that?<br>Which of the pictures is easy to understand, hard to understand, or not understood at all? Why?<br>For those who do not understand these pictures, have you ever tried to understand them? What has been done? Why is that so?	The probing questions (PQ.) were only used when discussions are stalled
	**Closing of the FGD**	At the end of the FGD, the participants were allowed to come up with additional remarks or suggestions

*****FGD focus group discussion; IQ, introductory question; KQ, Key question; PQ, probing question*
